# Analgesic comparison between topical irrigation (splash block) versus injection of lidocaine on the ovarian pedicle in canine ovariectomy

**DOI:** 10.1002/vms3.843

**Published:** 2022-05-25

**Authors:** Vincenzo Cicirelli, Giovanni Michele Lacalandra, Sandor Cseh, Daniela Mrenoshki, Edoardo Lillo, Francesco Paolo Bianchi, Giulio Guido Aiudi

**Affiliations:** ^1^ Department of Veterinary Medicine University of Bari “Aldo Moro” Bari Italy; ^2^ Department and Clinic of Obstetrics and Animal Reproduction University of Veterinary Medicine Budapest Hungary; ^3^ Department of Biomedical Science and Human Oncology University of Bari “Aldo Moro” Bari Italy

**Keywords:** analgesia, lidocaine, ovariectomy, splash block

## Abstract

**Objectives:**

The aim of this study was to compare the analgesic efficacy of topical irrigation versus injection of lidocaine on the ovarian pedicle to provide analgesia in bitches ovariectomy. In the current study were monitored: increased blood pressure, heart rate and respiratory rate to identify an acute intraoperative nociceptive response. These parameters were registered at six times during the surgical procedure: grasping of the ovary (time 1), dissection of the mesosalpinx (time 2), tightening of the first loop ligature (time 3), tightening of the second loop ligature (time 4), transection of the ovarian pedicle (time 5) and release of the ovary (time 6).

**Material and Methods:**

Forty healthy bitches were randomly assigned in two groups (n = 20) to receive topical irrigation (splash block) of 2% lidocaine (C group) on both ovarian pedicle (2 mg/kg each), or an equal volume of lidocaine was injected at the same sites (R group).

**Results:**

The results of the present study suggest that splash block may provide intraoperative analgesic effects equivalent to injection in the ovarian pedicle in dogs that have undergone ovariectomy. The lidocaine improved surgical analgesia during canine ovariectomy in both groups, and this action is not affected by the inoculation technique.

**Clinical significance:**

Pain management in veterinary patients is a crucial component of appropriate patient care. Therefore, the need for achieving safer anaesthesia for surgical intervention is gaining much attention. Ovariectomy is a common surgical procedure in bitches with medium level of pain. This study concluded that considering its relative simplicity, low cost, and safety, both techniques could be used in daily clinical practice.

## Graphical abstract

The aim of the study is to compare the analgesic efficacy of topical irrigation versus injection of lidocaine on the ovarian pedicle to provide analgesia in bitches ovariectomy. The results suggest that splash block may provide an effective intraoperative analgesic effects equivalent to injection in the ovarian pedicle.

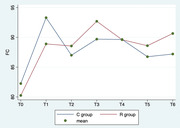

## 1 INTRODUCTION

Ovariectomy is a common surgical procedure in small animal practice, useful for reducing the stray population (Dongaonkar et al., 2019), obtaining therapeutic and behavioural benefits on neutered patients (Cicirelli et al., 2021), and is used as a clinical model for pain assessment studies (Wagner et al., 2008). In fact, despite the surgical simplicity of execution, ovariectomy is a surgery with medium level of pain and requires a good analgesic technique (Cicirelli et al., 2022, Gaynor & Muir, 2015). Many studies have been conducted to reduce the surgical pain, such as the use of local anaesthetic, to improve intraoperative analgesia with minimal systemic side effects, decreasing surgical stress response and reducing the patient's need of a rescue analgesia (Adin, 2011; Cicirelli et al., 2021, 2022). Surgical analgesia is important because pain induces various negative effects that prevent the patient from recovering (Cicirelli et al., 2022), such as a negative protein balance, reduced food intake, release of stress hormones, self‐mutilation and immunosuppression (Cicirelli et al., 2021, Gaynor, 1999). In several studies, local analgesics have been used in pain management in spayed domestic animals (Cicirelli et al., 2022, Cicirelli et al., 2022, Leoci et al., 2019). Splash block is an analgesic technique already described by several authors (Garwood et al., 2002, Tan et al., 2011, Wagner et al., 2008, Zilberstein et al., 2008) in domestic animals, which consists of an irrigation of lidocaine in the ovarian pedicle to improve local analgesia during ovariectomy. Furthermore, Grubb and Lobprise (2020) describe direct infiltration of the ovarian pedicle with lidocaine. To our knowledge, the use of lidocaine infiltrated on the canine pedicle during ovariectomy has not yet been evaluated or compared with other analgesic techniques. Although it is to be expected that additional local anaesthesia confers better analgesia, the aim of this study was to compare the analgesic efficacy of splash block versus the infiltration of lidocaine on the ovarian pedicle in bitches ovariectomy.

## 2 MATERIALS AND METHODS

### 2.1 Study design

This was a randomized clinical research study. All bitches were enrolled over a 6‐month period during 2021. The same team of surgeon performed all the procedures.

### 2.2 Animals

Forty bitches of various breeds presented for ovariectomy were involved in this study after obtaining informed owner consent. The female dogs were of good health, had no previous pathologies and were allocated to the very low aesthetic risk class (ASA 1). Sample size calculation was performed using G*Power for Windows Version 3.1.6 113 (Heinrich Heine Universität Düsseldorf, Germany) (Faul et al., 2007). Exclusion criteria included aggressiveness, underlying diseases and use of analgesics or anti‐inflammatory in the previous 30 days. Two days before surgery, patients underwent a comprehensive medical examination, including cardiothoracic auscultation, ECG, complete blood count (CBC), platelet count, total plasma proteins (TPP), serum creatinine, albumin, alanine aminotransferase (ALT), aspartate aminotransferase (AST), alkaline phosphatase (FA) and urea. The bitches were randomly (www.randomizer.org) assigned in two groups (n = 20) to receive topical irrigation (splash block) of 2% lidocaine (C group) on both ovarian pedicle (2 mg/kg each), or an equal volume of lidocaine was injected at the same sites (R group).

### 2.3 Pre‐surgery

Animals were submitted to solid and water fasting of 8 and 2 hours, respectively, prior to surgery. In both groups, the bitches were premedicated with intramuscular injections of dexmedetomidine (Dexdomitor®, Vetoquinol Italia SRL, Bertinoro, Italy) at dosage of 3 mcg/kg and methadone (Semfortan®, Eurovet Animal Health BV, Bladel, the Netherlands) at the dosage of 0.25 mg/kg, mixed in the same syringe (Cicirelli et al., 2022). The premedicants were administered into the lumbar epaxial muscles. After 20 min, a 20‐G venous catheter was inserted to start a standard maintenance fluid therapy (3 ml/kg/h of ringer with lactate). Propofol (Vetofol®, Esteve, Barcelona, Spain) at 2 mg/kg was administered intravenously to induce general anaesthesia. Orotracheal intubation was promoted, while anesthetic maintenance was performed with sevoflurane (EtSev 2,5%, SevoFlo®, Ecuphar Italia S.r.l., Milano, Italy), vaporized in 100% oxygen, in an open anesthesia system, always performed by the same anesthesiologist. During the perioperative period, the animals were continuously monitored through multiparametric monitoring heart rate, respiratory rate, non‐invasive blood pressure, oxygen haemoglobin saturation and body temperature (Cicirelli et al., 2022, Leoci et al., 2019).

### 2.4 Surgery procedure

All surgery were performed in 31 min (±5 min) from the start of the first skin incision to placement of the last skin suture. The quality of all procedures was approximately the same: the same surgeons performed all 40 procedures using a standardized surgical procedure. In C group, prior to manipulation of the ovarian pedicles, 2% lidocaine was dripped (Garwood et al., 2002, Tan et al., 2011, Wagner et al., 2008, Zilberstein et al., 2008) on the ovarian pedicle (2 mg/kg each) using a catheter urinary (splash block). In R group, 2% lidocaine was injected (Grubb & Lobprise, 2020) on the ovarian pedicle (2 mg/kg each) using a 2.5 mL syringe (23‐G). Following lidocaine application, surgical manipulation was stopped for 90 s (Wagner et al., 2008, Zilberstein et al., 2008). Before the first incision (T0), the hemodynamic parameters of all animals (preincisional values of heart, respiratory and blood non‐invasive pressure values) were recorded to evaluate pain responses to the surgical stimulus (Cicirelli et al., 2022, Leoci et al., 2019). These parameters were registered at six time points of the study: grasping of the ovary (T1), dissection of the mesosalpinx (T2), tightening of the first loop ligature (T3), tightening of the second loop ligature (T4), transection of the ovarian pedicle (T5) and release of the ovary (T6). In case of intraoperative increase of 30% of hemodynamic parameters respect the preincisional value, a bolus of fentanyl was administered i.v. (2 mcg/kg, Fentadon®, Eurovet Animal Health BV, Bladel, the Netherlands) (Campagnol et al., 2012). Before the end of the surgery, 0.2 mg of Meloxicam® (Metacam, Boehringer Ingelheim, Milan, Italy) was injected subcutaneously in all patients.

### 2.5 Data analysis

Compiled forms were entered into a database created with an Excel spreadsheet, and data analysis was performed using Stata MP17 software. Continuous variables were described as mean (standard deviation [SD]) and range, and categorical variables as proportions. The skewness and kurtosis test was used to evaluate the normality of continuous variables; all the continuous variables were normally distributed. The t student test for independent data was used to compare continuous variables between groups, the analysis of variance (ANOVA) for repeated measures test was used to compare continuous variables between groups and detection time; the Fisher's exact test was used to compare the proportions. For all tests, a two‐sided *p*‐value < .05 was considered statistically significant.

## 3 RESULTS

The population consisted of 40 bitches: 20 (50.0%) in the C group and 20 (50.0%) in the R group. The characteristics of the population according to the groups are presented in Table 1.

**TABLE 1:** Characteristics of the population according to group. Mean ± standard deviation and range of sample characteristics (age and weight) (C vs. R)

Variable	C (*n* = 20)	R (*n* = 20)	Total (*n* = 40)	*p*‐Value
Age (months)	18.5 ± 1.9	19.2 ± 2.2	18.8 ± 2.1	0.344
Weight (kg)	13.2 ± 0.5	13.4 ± 0.6	13.3 ± 0.5	0.256

**FIGURE 1:**
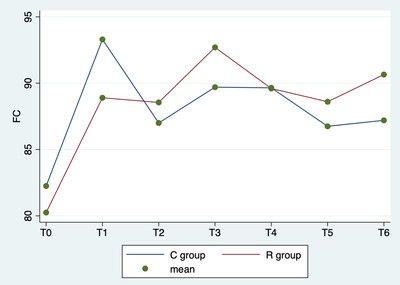
Repeated‐measures analysis of variance (ANOVA). Average heart rate values by group (C vs. R) and detection time

Repeated‐measures ANOVA showed significant differences in the comparison of heart rate among different times (p < 0.0001), but not between groups (p = 0.864) or in the interaction between time and group (p = 0.317; Figure 1).

**FIGURE 2:**
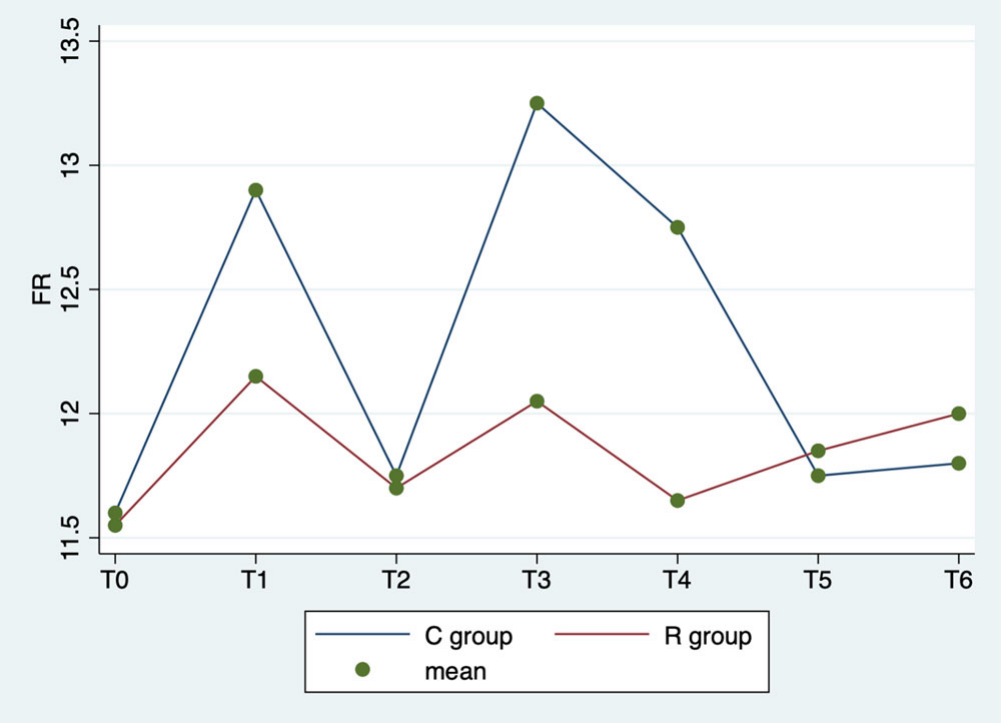
Repeated‐measures analysis of variance (ANOVA). Average respiratory rate by group (C vs. R) and detection time

Repeated‐measures ANOVA showed no significant differences in respiratory rate among various times (p = 0.151), between groups (p = 0.542) or in the interaction between time and group (p = 0.558; Figure 2).

**FIGURE 3:**
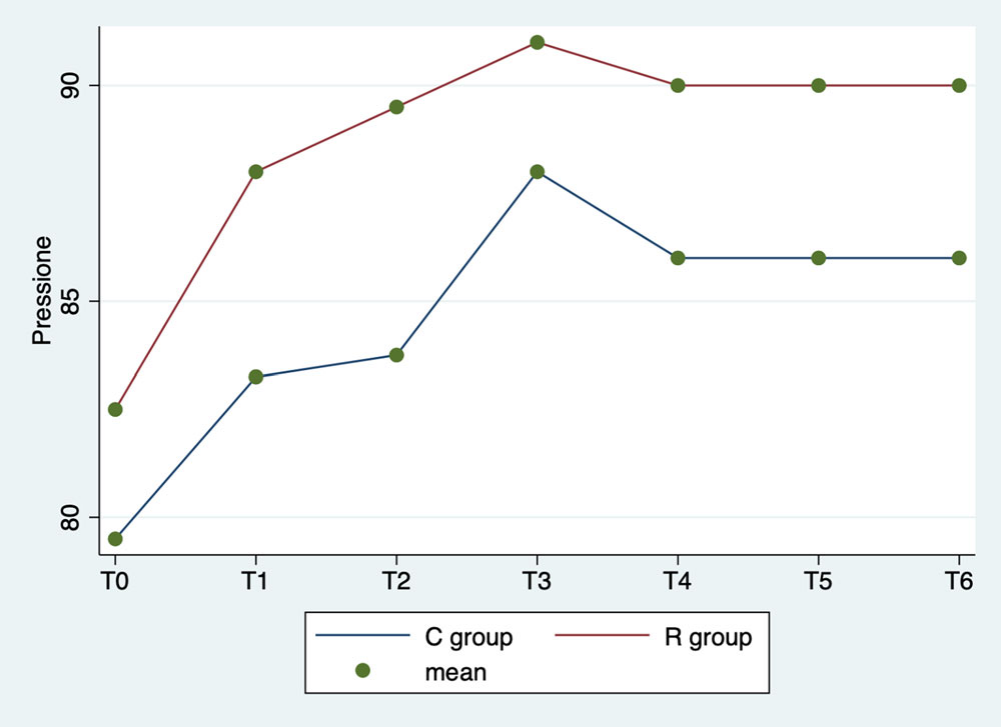
Repeated‐measures analysis of variance (ANOVA). Average blood pressure values by group (C vs. L) and detection time

Repeated‐measures ANOVA showed a significant difference in blood pressure values among the various times (p < 0.0001), but not between groups (p = 0.0001) or in the interaction between time and group (p = 0.900; Figure 3).

The proportion of dogs undergoing rescue analgesia according to group and detection time are described in Table 2.

**TABLE 2:** Proportion of dogs undergoing rescue analgesia by group (C and R) and detection time

Variable	C (*n* = 20)	R (*n* = 20)	Total (*n* = 40)	*p*‐Value
T1	1 (5.0%)	1 (5.0%)	2 (5.0%)	1.000
T3	0 (0.0%)	1 (5.0%)	1 (2.5%)	1.000
T4	1 (5.0%)	0 (0.0%)	1 (2.5%)	1.000

## 4 DISCUSSION

In this study, no significative side effects (e.g. haemorrhages, prolongation of time or cardiological alterations) were observed during the ovariectomy, and ovaries were removed without complications. No relevant hemodynamic problems were observed as a result of surgery. All dogs recovered from anaesthesia uneventfully and within 31 min (±5 min) after switching off the isoflurane vaporizer, and no immediate postoperative complications were observed during patient awakening. The results of this study show that all animals were ovariectomized under anaesthesia with a good surgery and analgesic technique. Indeed, multimodal analgesia, including drugs administered both systemically and locally, is considered the most effective approach to providing pain relief and has been widely accepted in veterinary medicine to control intraoperative pain (Campagnol et al., 2012). The combination of general and local anaesthesia to improve the analgesic protocol has been well documented for a variety of surgical procedures (Taylor & Robertson, 2004). The results of the present study suggest that splash block may provide intraoperative analgesic effects equivalent to injection in the ovarian pedicle in dogs that have undergone ovariectomy. In fact, lidocaine is absorbed quickly from ovarian tissue and block the ascending afferent input interfering with ion channels of the nerves of ovaries, which receive sympathetic fibres from the intermesenteric and the caudal mesenteric plexus and parasympathetic fibres from the vagus nerve (Katz et al., 1992). In this study, we choose to evaluate intraoperative hemodynamic parameters: blood pressure, heart rate and respiratory rate to identify a surgical nociceptive response. In literature, the correlation between these three parameters and surgical pain is commonly used (Weary et al., 2006). In fact, when the sympathoadrenal system is stimulated by a nociceptive stimulus, hemodynamic parameters increase (Zbinden et al., 1994, Eoh et al., 2018). In this study, there was no statistical difference between the R and C groups in the blood pressure, heart rate and respiratory rate. The trends observed in physiological parameters in two groups were statistically similar (Figures 1, 2 and 3). This study showed that the integration of lidocaine in routinely anaesthetic protocols is helpful to reduce the surgical nociceptive stimulus. Indeed, lidocaine when applied topically or infiltrated acts to stop the transmission of noxious stimuli (Torske & Dyson, 2000) and offers appropriate analgesia. Lidocaine has a fast onset (< 2 min) and short duration of action (1–2 h), so it is appropriate in ovarian manipulation (Fink & Schofield, 1970). The advantage is that this blockage desensitises the tissues innervated, providing excellent analgesia without side effects or respiratory depression (Collins et al., 2013). Carpenter et al. (2004) stated that lidocaine should be used in reproductive system surgical procedures, regardless of the administration technique (Feldman et al., 1989). In addition, considering the same low index of intraoperative rescue analgesia (Table 2) in both groups, it can be assumed that this treatment is effective enough to maintain physiological parameters at T1, T3 and T4. In fact, the grasping of the ovary, tightening of the first loop ligature and tightening of the second loop ligature are the moments correlated with greater autonomic stimulation in ovariectomy surgery (Cicirelli et al., 2022). Excessive local anaesthetic absorption can cause systemic toxicity (Feldman et al., 1989). An excessive volume of local anaesthetic could migrate cranially to the point of blockade of the nerves controlling the diaphragm, with pulmonary problems (Grubb & Lobprise, 2020, Sakura et al., 1998). The recommendation is not to exceed 20.8 ± 4 mg/kg and 6 ml for dog of lidocaine volume to prevent this complication (Grubb & Lobprise, 2020, Sakura et al., 1998). In this study, the dosage of lidocaine used was 4 mg/kg and maximum 3 ml for dog during the entire surgical procedure; therefore, it was not associated with systemic toxicity.

## 5 CONCLUSIONS

The present study showed that lidocaine administered by splash block or injection in the ovarian pedicle, during canine ovariectomy, is equivalent. This technique is not affected by the inoculation technique and confers satisfactory intraoperative analgesia during surgery procedure. Considering the cost, availability, and side effects of lidocaine, routine use of this local anaesthetic is considered desirable in bitch ovariectomy.

## CONFLICT OF INTEREST

The authors declare that they have no competing interests.

## AUTHOR CONTRIBUTIONS

Vincenzo Cicirelli: Formal analysis, Investigation, Methodology

Giovanni Michele Lacalandra: Conceptualization, Formal analysis, Funding acquisition

sandor Cseh: Data curation

Daniela Mrenoshki: CRediT contribution not specified

Edoardo Lillo: Investigation

Francesco Bianchi: Data curation

Giulio Aiudi: Supervision, Writing – review & editing

## ETHICS

The present study was performed in accordance with the ethical guidelines of the Animal Welfare Committee, and Institutional Review Board approval of the study was obtained (approval number: 02/21 [31/03/2021]). Animal procedures were performed following Directive 2010/63/EU of the European Parliament (Italian DL 26/2014).

### PEER REVIEW

The peer review history for this article is available at https://publons.com/publon/10.1002/vms3.843.

## Data Availability

The data presented in this study are available on request from the corresponding author.

## References

[vms3843-bib-0001] Adin, C. A. (2011). Complications of ovariohysterectomy and orchiectomy in companion animals. The Veterinary Clinics of North America. Small Animal Practice, 41(5), 1023–1039. 21889699 10.1016/j.cvsm.2011.05.004

[vms3843-bib-0002] Campagnol, D. , Teixeira‐Neto, F. J. , Monteiro, E. R. , Restitutti, F. , & Minto, B. W. (2012). Effect of intraperitoneal or incisional bupivacaine on pain and the analgesic requirement after ovariohysterectomy in dogs. Veterinary Anaesthesia and Analgesia, 39, 426–430. 22642413 10.1111/j.1467-2995.2012.00728.x

[vms3843-bib-0003] Carpenter, R. E. , Wilson, D. V. , & Evans, A. T. (2004). Evaluation of intraperitoneal and incisional lidocaine or bupivacaine for analgesia following ovariohysterectomy in the dog. Veterinary Anaesthesia and Analgesia, 31(1), 46–52. 14756753 10.1111/j.1467-2995.2004.00137.x

[vms3843-bib-0004] Cicirelli, V. , Accogli, G. , Caira, M. , Lacalandra, G. M. , & Aiudi, G. (2022). Use of ‘Aminogam Gel’ to fast the wound healing in dogs after the surgical curettage of injured penis. Veterinary Medicine and Science, [published online ahead of print, 2022 Mar 1], 10.1002/vms3.769 PMC9122447 35229984

[vms3843-bib-0005] Cicirelli, V. , Aiudi, G. G. , Mrenoshki, D. , & Lacalandra, G. M. (2022). Fentanyl patch versus tramadol for the control of postoperative pain in canine ovariectomy and mastectomy. Veterinary Medicine and Science, 8(2), 469–475. 34953046 10.1002/vms3.691 PMC8959330

[vms3843-bib-0006] Cicirelli, V. , Debidda, P. , Maggio, N. , Caira, M. , Lacalandra, G. M. , & Aiudi, G. G. (2021). Ultrasound‐Guided Funicular Block: Ropivacaine Injection into the Tissue around the Spermatic Cord to Improve Analgesia during Orchiectomy in Dogs. Animals, 11(5), 1275. 33925210 10.3390/ani11051275 PMC8146739

[vms3843-bib-0007] Cicirelli, V. , Debidda, P. , Maggio, N. , Caira, M. , Mrenoshki, D. , Aiudi, G. G. , & Lacalandra, G. M. (2021). Use of Spinal Anaesthesia with Anaesthetic Block of Intercostal Nerves Compared to a Continuous Infusion of Sufentanyl to Improve Analgesia in Cats Undergoing Unilateral Mastectomy. Animals, 11(3), 887. 33804684 10.3390/ani11030887 PMC8003676

[vms3843-bib-0008] Cicirelli, V. , Lacalandra, G. M. , & Aiudi, G. G. (2022). The effect of splash block on the need for analgesia in dogs subjected to video‐assisted ovariectomy. Veterinary Medical Science, 8(1), 104–109. 10.1002/vms3.637 PMC8788979 34647415

[vms3843-bib-0009] Cicirelli, V. , Macrì, F. , Di Pietro, S. , Leoci, R. , Lacalandra, G. M. , & Aiudi, G. G. (2022). Use of Contrast‐Enhanced Ultrasound of the Testes after Non‐Surgical Sterilization of Male Dogs with CaCl2 in Alcohol. Animals (Basel), 12(5), 577. 10.3390/ani12050577 35268146 PMC8909176

[vms3843-bib-0010] Collins, J. B. , Song, M. D. , & Mahabir, R. C. (2013). Onset and duration of intradermal mixtures of bupivacaine and lidocaine with epinephrine. Canadian Society of Plastic Surgeons, 21(1), 51–53. 10.1177/229255031302100112 PMC3891109 24431939

[vms3843-bib-0011] Dongaonkar, K. R. , Gulavane, S. U. , Chariar, V. M. , & Shelar, K. R. (2019). Laparoscopic ovariectomy in dogs in late gestation. BMC Veterinary Research, 15(1), 19. Published. 30621681 10.1186/s12917-018-1770-z PMC6325880

[vms3843-bib-0012] Eoh, K. J. , Lee, J. Y. , Nam, E. J. , Kim, S. , Kim, Y. T. , & Kim, S. W. (2018). Periumbilical infiltration of lidocaine with epinephrine for postoperative pain reduction in single‐port laparoscopic adnexal surgery. Journal of Obstetrics and Gynaecology, 38(8), 1135–1139. 30207501 10.1080/01443615.2018.1455079

[vms3843-bib-0013] Faul, F. , Erdfelder, E. , Lang, A. G. , & Buchner, A. (2007). G*Power 3: A flexible statistical power analysis program for the social, behavioral, and biomedical sciences. Behavior Researc Methods, 39, 175–191. 10.3758/bf03193146 17695343

[vms3843-bib-0014] Feldman, H. S. , Arthur, G. R. , & Covino, B. G. (1989). Comparative systemic toxicity on convulsant and supraconvulsant doses of intravenous ropivacaine, bupivacaine, and lidocaine in the conscious dog. Anesthesia and Analgesia, 69, 794–801. 2511782

[vms3843-bib-0015] Fink, G. , & Schofield, C. (1970). Innervation of the ovary in cats. Journal of Anatomy, 106(Pt 1), 191. 5461039

[vms3843-bib-0016] Garwood, S. , Reeder, M. , Mackenzie, I. , & Guillebaud, J. (2002). Tubal surface lidocaine mediates pre‐emptive analgesia in awake laparoscopic sterilization: A prospective randomized clinical trial. American Journal of Obstetrics and Gynecology, 186, 383–388. 11904595 10.1067/mob.2002.121079

[vms3843-bib-0017] Gaynor, J. S. (1999). Is postoperative pain management important in dogs and cats? Veterinary Medicine, 3, 254–257.

[vms3843-bib-0018] Gaynor, J. S. , & Muir, W. W. (2015). Handbook of veterinary pain management. Third edition. Missouri (USA), Elsevier, pp. 620.

[vms3843-bib-0019] Grubb, T. , & Lobprise, H. (2020). Local and regional anaesthesia in dogs and cats: Descriptions of specific local and regional techniques. Veterinary Medicine and Science, 6, 2218–234. 10.1002/vms3.218 PMC7196680 31965749

[vms3843-bib-0020] Katz, J. , Kavanagh, B. , Sabdler, A. , Nieremberg, H. , Boyland, J. , Friedlander, M. , & Shaw, B. (1992). Preemptive analgesia Clinical evidence of neuroplasticity contributing to postoperative pain. Anesthesiology, 77, 439–446. 1519781 10.1097/00000542-199209000-00006

[vms3843-bib-0021] Leoci, R. , Aiudi, G. , Cicirelli, V. , Brent, L. , Iaria, C. , & Lacalandra, G. M. (2019). Effects of intratesticular vs intraepididymal calcium chloride sterilant on testicular morphology and fertility in dogs. Theriogenology, 127, 153–160. 10.1016/j.theriogenology.2019.01.006 30708272

[vms3843-bib-0022] Sakura, S. , Sumi, M. , Yamada, Y. , Saito, Y. , & Kosaka, Y. (1998). Quantitative and selective assessment of sensory block during lumbar epidural anaesthesia with 1% or 2% lidocaine. British Journal of Anaesthesia, 81(5), 718–722. 10193282 10.1093/bja/81.5.718

[vms3843-bib-0023] Tan, C. Y. , Chen, H. C. , & Goh, C. F. (2011). Effect of splash block using Lidocaine in dogs undergoing ovariohysterectomy. In: 6th Proceedings of the Seminar on Veterinary Sciences, 11–14 Jan. 2011, Kuala Lumpur.

[vms3843-bib-0024] Taylor, P. , & Robertson, S. (2004). Pain management in cats – past, present and future Part 1. The cat is unique. Journal of Feline Medicine and Surgery, 6, 313–320. 15363763 10.1016/j.jfms.2003.10.003 PMC10822206

[vms3843-bib-0025] Torske, K. E. , & Dyson, D. H. (2000). Epidural analgesia and anesthesia. The Veterinary clinics of North America Small Animal Practice, 30, 859–874. 10932829 10.1016/s0195-5616(08)70011-1

[vms3843-bib-0026] Wagner, A. E. , Worland, G. A. , Glawe, J. C. , & Hellyer, P. W. (2008). Multicenter randomized controlled trial of pain‐related behaviors following routine neutering in dogs. Journal of the American Veterinary Medical Association, 233, 109–115. 18593318 10.2460/javma.233.1.109

[vms3843-bib-0027] Weary, D. M. , Niel, L. , & Flower, F. C. (2006). Identifying and preventing pain in animals. Applied Animal Behaviour Science, 100, 64–76.

[vms3843-bib-0028] Zbinden, A. , Petersen‐Felix, S. , & Thomson, D. (1994). Anesthetic depth defined using multiple noxious stimuli during isoflurane/oxygen anesthesia. II. Hemodynamic responses. Anesthesiology, 80, 261–267. 8311308 10.1097/00000542-199402000-00005

[vms3843-bib-0029] Zilberstein, L. F. , Moens, Y. P. , & Leterrier, E. (2008). The effect of local anaesthesia on anaesthetic requirements for feline ovariectomy. The Veterinary Journal, 178, 212–216. 10.1016/j.tvjl.2007.10.011 18036858

